# Electrochemical Biosensing Platforms for Rapid and Early Diagnosis of Crop Fungal and Viral Diseases

**DOI:** 10.3390/s26062004

**Published:** 2026-03-23

**Authors:** Yuhong Zheng, Li Fu, Jiale Yang, Shansong Gao, Haobo Sun, Fan Zhang

**Affiliations:** 1 Jiangsu Key Laboratory for Conservation and Utilization of Plant Resources, Institute of Botany, Jiangsu Province and Chinese Academy of Sciences (Nanjing Botanical Garden Mem. Sun Yat-Sen), Nanjing 210014, China; zhengyuhong@cnbg.net (Y.Z.); yangjiale@jib.ac.cn (J.Y.); gaoshansong@jib.ac.cn (S.G.); sunhaobo@jib.ac.cn (H.S.); 2 College of Materials and Environmental Engineering, Hangzhou Dianzi University, Hangzhou 310018, China

**Keywords:** plant pathogen diagnostics, nucleic acid hybridization sensing, impedimetric analysis, volatile biomarker monitoring, agricultural point-of-care technology

## Abstract

Crop fungal and viral diseases cause annual economic losses exceeding USD 150 billion globally, demanding rapid, sensitive, and field-deployable diagnostic technologies. This review critically evaluates recent advances in electrochemical biosensing platforms for early crop pathogen detection, focusing on immunosensors, genosensors, aptasensors, and VOC-based systems. Reported analytical performances demonstrate ultralow detection capabilities, including 0.3 fg mL^−1^ for viral coat proteins, 15 DNA copies for bacterial pathogens, 0.5 fg µL^−1^ RNA detection for viroids, and nanomolar-level VOC sensing (35–62 nM), with response times ranging from 2 to 60 min. Comparative analysis reveals that genosensors and aptasensors generally achieve the lowest LODs due to nucleic acid amplification or high-affinity recognition, while immunosensors provide robust protein-level specificity validated against ELISA. Volatile organic compound (VOC) sensors enable non-invasive, pre-symptomatic monitoring but face specificity challenges. Despite strong laboratory performance, practical adoption is limited by matrix-derived electrochemical interference, environmental instability of biorecognition elements, workflow complexity, and insufficient standardization across studies. Emerging innovations, including magnetic bead enrichment, nanoporous and graphene-based electrodes, microfluidic integration, AI-assisted impedance interpretation, and biodegradable substrates, are progressively addressing these bottlenecks. This review emphasizes that successful field translation requires holistic workflow engineering, matrix-matched validation, and harmonized performance metrics rather than incremental sensitivity improvements alone. By integrating analytical chemistry, nanomaterials engineering, and agricultural decision-support frameworks, electrochemical biosensing platforms hold significant potential to enable decentralized, rapid, and sustainable crop disease management.

## 1. Introduction

Crop fungal and viral diseases pose a persistent and devastating threat to global food security, with annual economic losses exceeding USD 150 billion worldwide due to reduced crop yields and quality degradation [1,2]. Traditional diagnostic methods, such as visual symptom observation, microbial culturing, and polymerase chain reaction (PCR), have long been the mainstays of crop pathogen detection [3], but they suffer from critical limitations: visual diagnosis relies on the appearance of overt symptoms, which often manifest only after irreversible crop damage has occurred; culturing methods are time-consuming, requiring days to weeks to yield definitive results [4]; and PCR-based techniques demand sophisticated laboratory equipment, trained personnel, and complex sample pre-processing, making them unsuitable for on-site, point-of-care (POC) applications in resource-limited agricultural settings [5].

**Figure 1:**
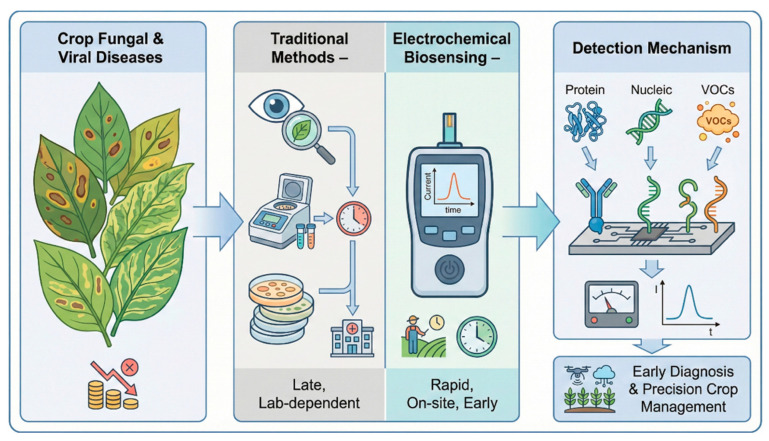
Comparison of traditional diagnostic methods and electrochemical biosensing strategies for crop fungal and viral disease detection. Traditional methods are typically labor-intensive, time-consuming, and laboratory-dependent, whereas electrochemical biosensing offers rapid, on-site, and early detection through the recognition of proteins, nucleic acids, and VOCs, thereby supporting precision crop disease management.

In response to these challenges, electrochemical biosensing platforms have emerged as a promising alternative for the rapid and early diagnosis of crop fungal and viral diseases [6]. These platforms leverage the unique advantages of electrochemical transduction, including high sensitivity, portability, low cost, and the ability to operate in complex matrices [7], enabling the detection of pathogen biomarkers, such as proteins, nucleic acids [8,9], and volatile organic compounds (VOCs) [10] at ultra-low concentrations, even before visible disease symptoms appear [11]. Over the past decade, advances in nanomaterial science [12], biomolecular recognition technology, and microfabrication [13] have further enhanced the performance of these biosensors, leading to a diverse range of platforms tailored to specific crop pathogen detection needs [14,15,16]. Importantly, the material used at the electrochemical interface is not only a passive support. It strongly influences electron-transfer efficiency, effective surface area, probe immobilization density, antifouling behavior, signal stability, and manufacturability. In crop disease diagnostics, commonly used material classes include gold nanostructures, graphene-derived materials, carbon nanotubes, metal oxides, mesoporous silica films, and emerging framework-based composites. Their value is application-dependent: noble metal and carbon nanomaterials often improve sensitivity and surface loading, whereas nanochannel and polymeric architectures can better control matrix interference, and low-cost paper-compatible substrates may be more attractive for field deployment even when their absolute analytical sensitivity is lower. Figure 1 provides a conceptual overview of the role of electrochemical biosensing platforms in addressing the limitations of conventional diagnostic strategies for crop fungal and viral diseases.

This review aims to provide a critical analysis of the current state of electrochemical biosensing platforms for the early detection of crop fungal and viral diseases. It will first classify the main types of electrochemical biosensors and their underlying detection mechanisms and then evaluate their performance metrics and inherent limitations through a critical examination of the recent peer-reviewed literature. The review will also explore the controversial perspectives surrounding the field application of these platforms, highlighting the key debates among researchers regarding their practical viability in agricultural settings. Finally, it will outline emerging trends and future directions for the development of next-generation electrochemical biosensors that can address the unmet needs of global crop disease management.

## 2. Classification and Mechanisms of Electrochemical Biosensing Platforms for Crop Pathogen Detection

Electrochemical biosensors for crop pathogen detection are broadly classified based on their biomolecular recognition elements and transduction mechanisms, including immunosensors, genosensors, aptasensors, and VOC-sensing electrochemical platforms. Each class of sensor exhibits unique advantages and is suited to different types of pathogen biomarkers and detection scenarios. To facilitate direct cross-platform comparison, Table 1 summarizes representative electrochemical biosensing studies discussed in this section, with emphasis on detection method, analyte, LOD, LOQ, and linear range.

### 2.1. Electrochemical Immunosensors

Electrochemical immunosensors exploit the high affinity of antigen–antibody recognition to convert pathogen-derived proteins (e.g., capsid/coat proteins, nucleocapsid proteins, fungal antigens) into quantifiable electrochemical outputs such as current, potential, or impedance shifts. In crop protection, the value proposition is not merely “sensitivity” but whether the format can tolerate complex plant matrices, support early-stage (often low-titer) detection, and remain deployable outside a centralized laboratory without sacrificing analytical specificity.

**Figure 2:**
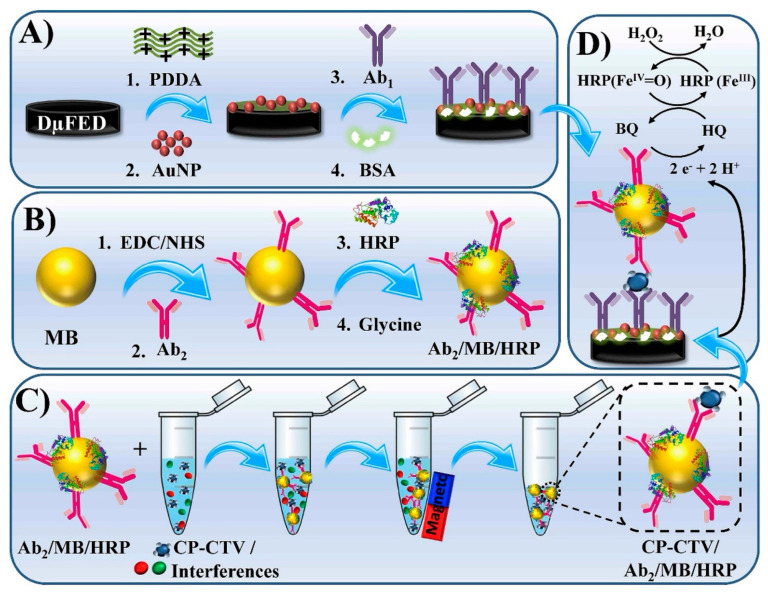
Schematic illustration. (**A**) Step of the surface modification of 8 working electrodes with PDDA, AuNP and monoclonal antibodies (Ab_1_). (**B**) Immunoconjugate preparation containing magnetic beads decorated with polyclonal antibodies and horseradish peroxidase enzymes. (**C**) Coat protein *Citrus tristeza virus* capture using immunoconjugate. (**D**) Sandwich immunoassay formation and electrochemical analysis [17].

A representative virus-targeting format is the disposable microfluidic electrochemical device (DμFED) proposed by Freitas et al. [17] for Citrus tristeza virus (CTV), one of the most economically consequential citrus diseases worldwide. Their approach combined magnetic bead-assisted capture/separation with a microfluidic array of immunosensors, using an HRP-labeled sandwich architecture and amperometric readout driven by a hydroquinone/H_2_O_2_ catalytic redox cycle (Figure 2). Importantly, this is not a “nanomaterial amplification” story alone; it is a workflow design that explicitly addresses plant sample heterogeneity by moving part of the recognition burden onto magnetic enrichment. The method exhibited a wide linear range (1.95–10.0 × 10^3^ fg mL^−1^) and an ultralow LOD (0.3 fg mL^−1^) and was validated in healthy versus infected plant samples with agreement to ELISA. The analytical strength here is the coupling of immunocapture, washing, and electrochemical transduction in a disposable format, which directly targets the operational constraints of field diagnosis rather than optimizing only electrode performance. That said, the use of HRP and substrate chemistry introduces reagent logistics (stability, temperature dependence, and timed development), which can become the true bottleneck in rugged field deployment unless lyophilization or closed-cartridge fluid handling is engineered.

A contrasting philosophy is “label-free” virus detection on a functionalized electrode, where the signal is generated by altered interfacial electron transfer rather than enzymatic catalysis. Haji-Hashemi et al. [18] reported a label-free immunosensor for Fig mosaic virus (FMV) by immobilizing antibodies against the viral nucleocapsid on a mixed-thiol self-assembled monolayer (11-mercaptoundecanoic acid and 3-mercaptopropionic acid) on gold, followed by carbodiimide coupling. Detection was performed via voltammetric interrogation (DPV) using ferri/ferrocyanide as a redox probe. The sensor achieved a 0.1 nM–1 μM working range with a 0.03 nM detection limit and was demonstrated in real samples without PCR amplification. The appeal of this class lies in its simpler workflow because it does not require enzyme labels and uses fewer reagents. However, the trade-off is that label-free responses can be more vulnerable to nonspecific adsorption and matrix-driven interfacial fouling, particularly in crude leaf extracts rich in phenolics, polysaccharides, and particulates. Consequently, blocking chemistry and surface passivation, often treated as minor experimental details, become decisive determinants of false positives/negatives and inter-assay reproducibility.

The recent work of Rezaei et al. [19] illustrates how these two philosophies, label-free simplicity versus label-based sensitivity, can be compared within the same study framework for a high-concern emerging virus, Tomato brown rugose fruit virus (TBRFV). They built direct and sandwich immunoassays on a nanoporous gold electrode to increase the electroactive area and support high-density capture. Both formats achieved linear detection across 10–10^5^ fg mL^−1^; the direct assay delivered an LOD of 1.14 fg mL^−1^ (65.14 aM), while the sandwich assay, further amplified with an HRP-labeled secondary antibody and enzymatic substrate chemistry, achieved 1.06 fg mL^−1^ (60.57 aM). They also demonstrated discrimination against other viruses and detection in leaf and seed extracts. A critical point, however, is that the numerical LOD advantage of the enzyme-amplified sandwich format was modest in this case, suggesting that the nanoporous electrode architecture and immunocapture efficiency dominated the analytical gain, while enzymatic amplification mainly improved signal robustness at ultra-low concentrations. This is a useful reminder for crop diagnostics: if sample extraction and antigen accessibility are limiting, chasing ever-lower instrument LOD can yield diminishing practical returns unless paired with improved sampling and preconcentration strategies.

Beyond well-known horticultural viruses, Verma et al. [24] developed an electrochemical immunosensor for Potato aucuba mosaic virus (PAMV), emphasizing how the shape and identity of the nanomatrix influences antibody presentation and therefore immunorecognition efficiency. They compared spherical AuNP-based and reduced graphene oxide (rGO) nanosheet-based immunoelectrodes, reporting LODs of 0.96 × 10^−12^ M (AuNP) and 0.065 × 10^−12^ M (rGO), and validated performance in PAMV-positive controls spiked into potato leaf samples as well as in real leaf samples. The critical insight is not merely that “rGO is better” but that extended 2D supports can alter effective antibody orientation, surface crowding, and local mass transport near the interface, factors that can dominate signal response in label-free or minimally amplified configurations. The practical challenge is that nanomatrix-dependent performance can be hard to generalize across pathogens and sample types; thus, studies should avoid over-claiming universality unless multi-matrix validation (multiple cultivars, different infection stages, and interfering plant proteins) is shown.

Electrochemical immunosensors are equally relevant for fungal diseases, where early detection is complicated by heterogeneous antigen distribution and the fact that many assays target complex fungal antigens rather than a single conserved viral capsid. Fernández-Baldo et al. [25] reported an SPCE immunosensor modified with carbon nanotubes for Botrytis cinerea determination in apple tissues via a competitive immunoassay strategy. The assay quantified bound antibody through HRP-labeled secondary antibodies using a catechol mediator system and the electrochemical signal was read at −0.15 V. Notably, they compared electrochemical detection against ELISA, reporting markedly improved detection capability for the electrochemical method (LOD 0.02 μg mL^−1^) versus ELISA (10 μg mL^−1^), with an assay time of ~30 min and intra-/inter-assay CVs below 7%. This case is instructive because it demonstrates an analytically strong bridge to plant tissue testing rather than buffer-only validation; however, competitive formats can be more sensitive to reagent lot variability (antigen immobilization consistency, antibody affinity drift) and may require more careful calibration management for long-term field use than direct/sandwich assays.

For soybean rust, an archetypal fungal disease where early identification is crucial, Mendes et al. [26] developed a label-free impedimetric immunosensor targeting Phakopsora pachyrhizi in soybean leaf extract, aiming specifically at detection before visible symptoms appear. Their report highlighted optimization of experimental conditions and surface blocking to reduce nonspecific adsorption and they reported a limit of detection of 385 ng mL^−1^. The conceptual value of this work is the explicit focus on early-stage diagnosis in complex leaf matrices using EIS, which is inherently sensitive to interfacial changes. The limitation, common to many EIS-based label-free fungal assays, is interpretability: impedance shifts can arise from target binding but also from matrix adsorption, ionic strength variation, or changes in the dielectric environment. Unless robustness is demonstrated across multiple field-relevant confounders (cultivar differences, leaf age, pesticide residues, and co-infections), there is a risk of “good lab curves” failing under agronomic reality.

Across these examples, the material platform emerges as a decisive factor linking analytical performance to deployability. Gold-based interfaces are advantageous for well-controlled probe immobilization and reproducible electrochemical readout, especially when thiolated capture layers are needed, but flat Au surfaces do not necessarily maximize signal unless complemented by surface nanostructuring. Nanoporous gold partially solves this by increasing electroactive area and capture density, which helps explain why the TBRFV assays reached attomolar sensitivity with only a modest additional gain from enzyme amplification. Carbon nanomaterials, including CNTs and graphene derivatives, are valuable because they enhance conductivity and accessible surface area, yet their benefit depends strongly on surface passivation; otherwise, the same high-area interface that improves response may also increase fouling in crude plant extracts. Material choice also affects the type of robustness achieved: magnetic bead-assisted and microfluidic systems improve pre-analytical clean-up, nanochannel films improve anti-interference control at the interface, and paper-compatible composites improve disposability and cost. Accordingly, the best material is not the one with the highest intrinsic conductivity but the one that addresses the dominant limitation of the intended crop-diagnostic workflow.

### 2.2. Electrochemical Genosensors

Electrochemical genosensors detect pathogen nucleic acids (DNA or RNA) through sequence-specific hybridization between a target strand and a complementary probe immobilized on an electrode surface, with hybridization converted into a measurable faradaic or non-faradaic electrochemical change. In crop disease diagnostics, their practical advantage is temporal: nucleic acids can be detectable at very low abundance before strong protein biomarker accumulation, but this advantage is only realized if the assay chemistry can withstand crude plant matrices and if the workflow does not reintroduce “laboratory dependence” via complex extraction and temperature control.

**Figure 3:**
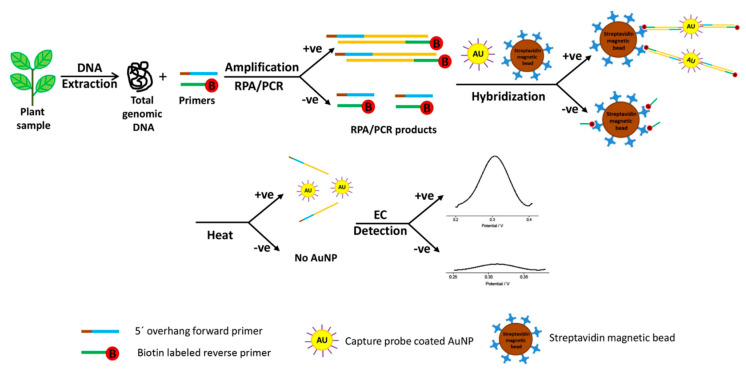
Schematic illustration of the electrochemical bioassay for plant pathogen DNA detection [9].

A widely cited isothermal paradigm is the colloidal gold nanoparticle (AuNP) electrochemical genosensor reported by Lau et al. [9] for *Pseudomonas syringae* detection on disposable SPCEs, in which pathogen DNA was first amplified by RPA and the resulting short amplicons were then detected electrochemically using AuNP-tagged probes and DPV readout (Figure 3). This distinction is important for field relevance: the electrochemical step does not directly interrogate native long genomic DNA in crude plant extracts but rather a pre-processed amplification product. Accordingly, the reported 15-copy sensitivity reflects the performance of an integrated amplification–electrochemical workflow, not a separation-free direct DNA assay. In plant samples, the dominant practical challenge is therefore not only electrode sensitivity but whether sample preparation can generate accessible target nucleic acids with adequate purity and reproducibility under non-laboratory conditions. By using an isothermal amplification front-end, the platform shifts the limiting step away from interfacial signal transduction and toward primer/probe design and contamination control, an important but sometimes under-discussed trade-off because RPA’s high sensitivity can also amplify trace carryover contamination if cartridge design and workflow separation are not engineered.

A complementary direction avoids enzymatic amplification altogether by leveraging high-affinity hybridization and highly responsive impedance platforms. Lobato et al. [20] developed a label-free impedimetric genosensor for Citrus Bark Cracking Viroid (CBCVd) detection directly from total RNA hop extracts, using probe capture assisted by streptavidin–agarose beads and electrochemical impedance spectroscopy (EIS) readout. Their optimized system achieved an LOD of 0.5 fg µL^−1^ (reported as 5.5 fmol L^−1^) with a one-hour incubation on denatured total RNA extract, explicitly aiming to eliminate the operational burden of amplification. This case is valuable for crop deployment because it confronts a central field constraint: amplification can be the most fragile step under variable temperature, operator skill, and cross-contamination risk. However, amplification-free EIS assays are intrinsically more sensitive to matrix-driven interfacial effects (ionic strength, nonspecific adsorption, viscosity, and plant phenolics), meaning that assay robustness depends heavily on antifouling surface chemistry and stringent negative controls across cultivar and tissue variability; without that breadth of validation, a low buffer-based LOD can overstate field reliability.

For fungal pathogens, genosensor development often targets virulence-associated genes or conserved internal transcribed spacer (ITS) regions, but the choice of target strongly influences both specificity and regulatory utility (species discrimination versus pathogenic race discrimination). Zhang et al. [27] designed an electrochemical nucleic acid sensor for *Fusarium oxysporum* f. sp. cubense Tropical Race 4 (Foc TR4) by targeting the dicer-like (DCL) gene, using a signal strategy described as an “on–off” design with DNA architectures and electrochemical reporting. The study frames the assay as a route to rapid containment by enabling sensitive and specific detection of a high-impact banana wilt agent at the molecular level. Conceptually, race-level targeting is a major strength because management decisions (quarantine, eradication, movement restrictions) depend on discriminating TR4 from less aggressive strains. The analytical risk is that virulence-linked genes can show sequence variation across geographic isolates; therefore, assays must demonstrate inclusivity (multiple isolates) and exclusivity (near-neighbor Fusarium and soil microbiome DNA) to avoid false negatives/positives in diverse agroecological settings.

Another fungus-focused example addresses Ustilaginoidea virens, the agent of rice false smut. Rana et al. [28] reported a graphene-based electrochemical DNA biosensor for U. virens, aiming to replace time-consuming and equipment-intensive conventional diagnostics with a nanomaterial-supported electrochemical hybridization platform. Graphene’s role is not merely “conductive enhancement”: 2D materials can provide high probe loading density and improved interfacial electron transfer, but they can also increase nonspecific adsorption if not carefully passivated. Therefore, the critical determinant becomes whether the surface chemistry yields reproducible probe orientation and stable baseline signals over repeated measurements, particularly in rice panicle/seed-associated extracts where polysaccharides and proteins can be abundant. Studies in this class are strongest when they report performance in realistic matrices and provide stability/reproducibility statistics over storage and repeated use.

Oomycete detection illustrates how genosensors can be designed for high-specificity genomic signatures. Franco et al. [29] developed a DNA-based electrochemical nanobiosensor for Phytophthora palmivora (black pod rot in cacao), designing oligonucleotide probes based on the ITS sequence of field isolates and forming a sandwich hybrid structure between capture probe, target genomic DNA, and signaling probe to generate the electrochemical readout. This approach is diagnostically meaningful because ITS regions are widely used for species discrimination in plant pathology, and a well-chosen probe set can provide specificity without the complexity of full amplification workflows. The practical challenge is not fragmentation alone but the need to convert heterogeneous field material into target nucleic acids that can hybridize efficiently at the electrode. In many electrochemical DNA assays, this still requires extraction, denaturation, amplification, magnetic separation, or preparation of short accessible targets before measurement. Franco et al. demonstrated that genomic DNA from cacao samples could be analyzed electrochemically, but the assay still relied on sandwich hybrid formation and washing steps rather than direct analysis of untreated field material. Therefore, electrochemical detection of pathogen-derived DNA fragments is not useless in real settings, but its utility depends on workflow integration; without simplified sample-to-answer processing, the requirement for nucleic acid preparation remains a major barrier to routine field deployment.

A mechanistically distinct platform is non-faradaic DNA sensing on interdigitated microelectrodes, which can be attractive for portable instrumentation because it avoids adding redox probes and can be read with low-power electronics. Patel et al. [30] reported a label-free, non-faradaic impedimetric DNA biosensor for the soil-borne bacterial phytopathogen Ralstonia solanacearum, using micro-sized gold interdigitated electrodes and a universal 30-mer probe (lpxC4) immobilized with mercaptohexanol, achieving a reported LOD of 0.1 ng µL^−1^ and selectivity against other common soil pathogens. Although this is a bacterial case, it is highly instructive for crop genosensor design because it shows how electrode geometry and capacitive sensing can produce sensitive responses without enzymatic steps. The limitation is interpretability and susceptibility to environmental variability: non-faradaic impedance signals can drift with temperature, ionic composition, and nonspecific adsorption. To be credible for field use, such systems must include rigorous baseline correction strategies, standardized sample dilution buffers, and, ideally, internal controls to distinguish true hybridization from matrix-induced dielectric changes.

These cases show that the real engineering question is not whether electrochemistry can detect short pathogen-derived DNA fragments but whether the overall assay can access those fragments from crude field samples without burdensome sample preparation. Isothermal amplification (e.g., RPA) can deliver extremely low copy-number detection and symptom-preceding diagnosis, but it shifts failure modes toward contamination management, primer specificity, and reagent stabilization. Amplification-free EIS platforms can simplify field workflows and reduce contamination risk, yet they demand exceptionally well-designed antifouling interfaces and extensive matrix-robustness validation because the measurement is fundamentally an interfacial phenomenon influenced by many non-target factors. Target selection is not a cosmetic detail: conserved regions (ITS) favor species detection and broad inclusivity, while virulence/race-associated genes (e.g., DCL in Foc TR4) can be more actionable but require stronger evidence of inclusivity across isolates to avoid geographically biased false negatives. Finally, nanomaterials (graphene, AuNP tags) and microelectrode architectures can boost signal and miniaturization potential, but they can also amplify nonspecific adsorption and inter-assay variability unless surface chemistry and blocking are treated as first-order design variables rather than routine steps.

### 2.3. Electrochemical Aptasensors

Electrochemical aptasensors use aptamers, short, single-stranded DNA or RNA ligands that fold into target-binding conformations, as biorecognition elements. In crop disease diagnostics, their appeal is not only cost and synthetic accessibility but also chemical programmability: aptamers can be end-labeled with redox reporters, engineered into structure-switching motifs, or paired with nucleic acid amplification circuits in ways that antibodies generally cannot. A notable crop-focused example is the aptamer-based electrochemical biosensor developed by Krivitsky et al. [21] for the detection of airborne soybean rust fungi urediniospores. The device used a porous 3D carbon electrode matrix to physically collect spores from air and then applied a biotinylated aptamer and a streptavidin–alkaline phosphatase conjugate to generate an electroactive enzymatic product measurable by DPV. Under their test conditions, the platform could detect ~100–200 spores cm^−2^ within minutes, highlighting a workflow advantage that is often more important than a low numerical LOD: direct coupling of sampling (air capture) and molecular recognition (aptamer binding) on the same electrode.

To broaden beyond airborne spores, electrochemical aptasensors have also been deployed against fungal infection–linked molecular signatures in crops, especially mycotoxins. While mycotoxin presence is not always synonymous with active field infection (and can reflect post-harvest growth), toxin monitoring is tightly connected to fungal disease impact because it captures the downstream consequence most relevant to food/feed safety and trade. For example, Wu et al. [31] introduced a dual-signal (ratiometric) electrochemical aptasensor for aflatoxin B1 (AFB1), where the ratiometric design was used explicitly to reduce susceptibility to electrode-to-electrode variability and background drift, two chronic problems for point-of-need electrochemistry in complex matrices. The methodological message is important for crop applications: when the matrix is inconsistent (grain extracts vary widely in ionic strength, oils, and particulates), ratio readouts can be more robust than single-channel currents, even if the “best-case” LOD is not dramatically different.

A different engineering route toward field robustness is the paper-based format. Zhang et al. [27] reported an electrochemical “signal on/off” paper-based aptasensor for ochratoxin A (OTA) using MXene–Au as the sensing substrate and catalytic amplification (Pt nanoparticles anchored on hollow NiCo layered double hydroxides). The assay leverages aptamer hybridization/dehybridization steps to switch signal states and provides an electrochemical response compatible with low-cost devices. In crop monitoring, paper devices matter because they reduce cleaning, cross-contamination, and operator dependence. The critical caveat is that multilayer catalytic amplification, while impressive on paper, can be reagent- and fabrication-sensitive: slight variation in nanozyme activity, probe density, or drying conditions can introduce lot-to-lot differences that may dominate performance when moved out of controlled lab workflows.

Patulin (PAT), a toxin associated with fungal spoilage of apples and related products, illustrates how aptamer interface engineering can address binding efficiency and steric accessibility. He et al. [32] built an electrochemical aptasensor on tetrahedral DNA nanostructures (TDNs) to present the aptamer in a more upright, spatially defined architecture, combined with thionine-labeled Fe_3_O_4_ nanoparticles on rGO for signal amplification. Their approach underscores a recurring aptasensor reality: for many targets, especially small molecules, the limiting step is not electron transfer but productive aptamer folding and target access at the interface. The strength of TDN-type architectures is improved interfacial reproducibility and reduced surface crowding; the weakness is that they increase fabrication complexity and cost, which can erode the “aptamers are cheaper than antibodies” argument unless the added complexity is justified by markedly improved stability across real sample sets.

**Figure 4:**
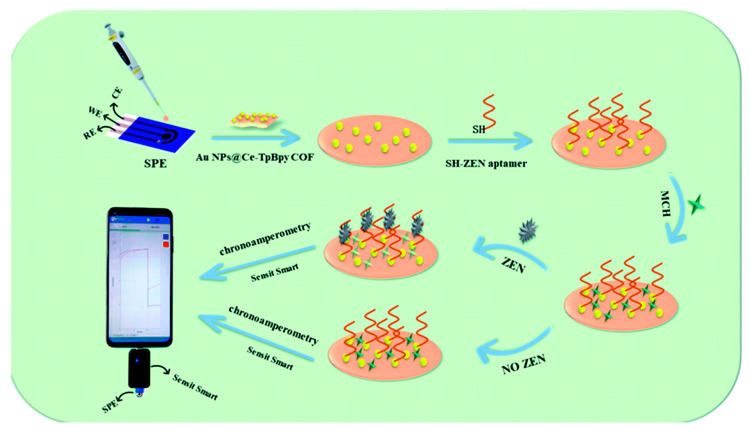
Preparation process of ZEN aptasensor [33].

Zearalenone (ZEN), a Fusarium-associated mycotoxin relevant to cereals, has also been widely used as a testbed for portable electrochemical aptasensing. Chen et al. [33] demonstrated an aptasensor designed for a compact “U-disk” electrochemical workstation, using a catalytic material (Ce-doped covalent organic framework, Ce–TpBpy COF) and chronoamperometric readout to quantify ZEN (Figure 4), explicitly aiming at convenience and deployability rather than only analytical sensitivity. This is a useful contrast to highly engineered ratiometric and DNA-circuit designs: for crop screening, instrument compatibility and operator simplicity can be more decisive than achieving an extra order of magnitude lower LOD. However, chronoamperometric catalytic schemes can be sensitive to dissolved oxygen, buffer composition, and temperature, variables that fluctuate in field or warehouse environments, so studies that do not report robustness testing across these factors can overestimate real-world reliability.

Another practical design pattern that maps well to crop extracts is nanochannel-based permselectivity, where the electrode is coated with a film that enriches a redox probe while restricting interferents. A representative example is a label-free electrochemical aptasensor for AFB1 based on vertically ordered mesoporous silica nanochannel films (VMSF) functionalized with an AFB1 aptamer, using Ru(NH_3_)_6_^3+^ as the signal probe. Here, the nanochannels provide cationic enrichment of the probe while the aptamer–target interaction gates transport, producing an amplified response [33]. The conceptual advantage in agricultural matrices is interference management: plant/food extracts contain diverse electroactive species and macromolecules that can foul electrodes. A nanochannel “filter + preconcentrator” can improve tolerance to such backgrounds. The main limitation is that film integrity and fouling over time become performance-critical; if the nanochannels clog or crack under handling, the sensor response may drift in ways that mimic target binding.

**Figure 5:**
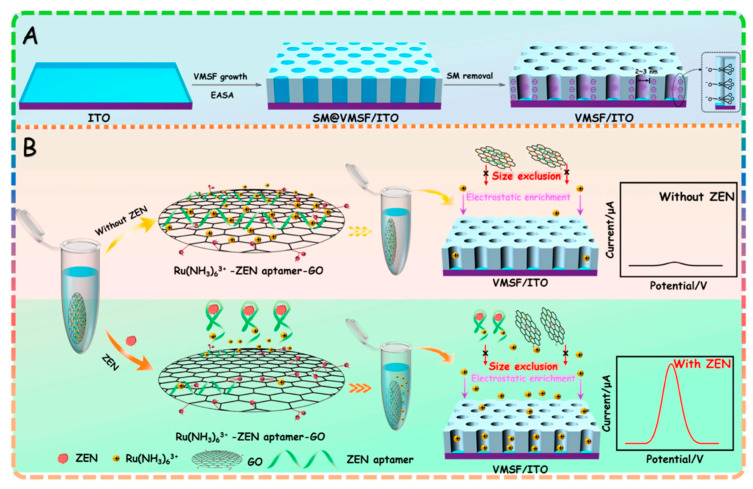
(**A**) Scheme of VMSF/ITO electrode prepared by using EASA method. (**B**) Schematic illustration of the homogeneous electrochemical aptasensor for the determination of ZEN [34].

Finally, homogeneous electrochemical aptasensing strategies can reduce dependence on surface assembly quality by moving part of the recognition/amplification into solution. Huang et al. [34] reported a homogeneous electrochemical aptasensor for ZEN using a nanocomposite probe and a silica nanochannel film (Figure 5), representing an approach that tries to combine solution-phase recognition benefits with electrode-phase signal management. From a critical standpoint, homogeneous designs can improve repeatability across electrodes and reduce sensitivity to minor immobilization defects, but they may add steps (mixing/incubation) that complicate true “in-field” workflows unless integrated into closed cartridges.

There are critical implications for crop fungal/viral diagnosis. Across these examples, the most meaningful differentiator is not whether the sensor uses DPV, SWV, or amperometry but where the assay places complexity. The soybean rust spore system achieves field relevance by integrating sample collection with recognition at the electrode level, which is directly aligned with early warning needs. Mycotoxin aptasensors, in contrast, often excel analytically but can drift away from “disease diagnosis” toward “contamination screening.” That is not a flaw—crop health management increasingly spans pre-harvest and post-harvest control—but it changes how performance should be judged: robustness to extraction variability, false positive control in diverse cultivars, and correlation to actionable thresholds become more important than the lowest LOD.

Aptamers offer real advantages over antibodies, including greater chemical stability, simpler synthesis, and easier modification; however, these advantages do not automatically translate into field-ready sensors. Many aptasensors show outstanding sensitivity in buffer yet provide limited evidence of matrix generalization (different crop varieties, infection stages, co-contaminants). Amplification-heavy constructs (nanozymes, DNA nanostructures, ratiometric dual labels) can reduce analytical noise, but they also increase susceptibility to manufacturing and storage variability. Conversely, “anti-interference” architectures such as nanochannels can improve tolerance to complex extracts, but long-term stability and fouling must be proven under realistic handling and storage. For viral crop diseases specifically, the strongest translational opportunity remains aptamers against viral capsid/nucleocapsid proteins coupled with disposable electrochemical formats; however, compared with fungal toxin work, there are still fewer well-validated electrochemical aptasensor case studies in plants, and future progress likely depends on expanding high-quality SELEX libraries against plant-virus proteins and demonstrating performance in crude leaf/sap matrices under field constraints.

### 2.4. Electrochemical VOC Sensors

Electrochemical VOC sensors target volatile organic compounds emitted by crops as a consequence of pathogen metabolism and host defense reprogramming, translating VOC–electrode interactions into quantifiable current- or impedance-based readouts [10]. Compared with protein or nucleic acid sensing, VOC sensing is attractive for “pre-symptomatic” screening because emissions can shift within hours to days after infection and be sampled non-destructively from headspace. At the same time, VOC-based diagnosis is intrinsically more ambiguous than sequence-based assays: many VOCs are stress markers rather than pathogen identifiers and their abundance is strongly shaped by cultivar, phenology, temperature, humidity, and soil status. The most convincing electrochemical VOC studies therefore (i) justify why a given VOC is mechanistically linked to infection, (ii) demonstrate analytical selectivity under realistic interferent backgrounds, and (iii) validate performance in plant extracts or contaminated air rather than only in neat solutions.

A representative pathogen-linked target is p-ethylguaiacol, reported as a fingerprint compound in the volatile signature of fruits and plants infected with Phytophthora cactorum. Fang, Umasankar, and Ramasamy constructed disposable SPCEs modified with SnO_2_ or TiO_2_ nanoparticles and quantified p-ethylguaiacol using voltammetric electroanalysis (CV/DPV), reporting high sensitivity (174–188 μA cm^−2^ mM^−1^) and nanomolar-level detection limits (35–62 nM) with good repeatability (RSD ≈ 2.48–4.85%) and limited interference from other common plant volatiles [23]. This study is technically important because it demonstrates that relatively inexpensive metal oxide modifiers can replace noble metal electrodes for oxidative VOC readouts, reducing cost barriers for field deployment. Conceptually, however, the “biological specificity” still hinges on the assumption that p-ethylguaiacol is sufficiently unique to that infection context; in practice, phenolic volatiles can also arise from microbial consortia on damaged tissue, so sampling protocols (wounding control, storage history) become as critical as electrode chemistry.

A second, widely discussed plant stress volatile is methyl salicylate (MeSA), a systemic acquired resistance signal that increases under biotic challenges. Umasankar and Ramasamy showed that MeSA can be detected electrochemically on AuNP-modified SPCEs in alkaline electrolytes, with AuNP–SPCE providing substantially higher sensitivity than unmodified SPCE and retaining most of its MeSA response even in the presence of abundant green leaf volatile interferents (e.g., cis-3-hexenol, hexyl acetate, cis-hexenyl acetate) [35]. The authors further demonstrated the feasibility of plant extract matrices, which is a critical step beyond buffer-only characterization [35]. From a critical standpoint, MeSA highlights a core interpretability issue in VOC diagnosis: MeSA is strongly associated with plant defense signaling, but it is not pathogen-exclusive. A MeSA-only sensor can be excellent for “stress alarm” functions (early warning, triage), yet it is weaker as a standalone diagnostic for differentiating fungal vs. viral etiologies unless paired with additional markers.

To push MeSA sensing toward stronger biochemical selectivity, Fang and co-workers developed a bi-enzyme cascade electrode (salicylate hydroxylase + tyrosinase) immobilized on CNT-modified electrodes and implemented amperometric detection through enzymatic conversion pathways, achieving LOD ≈ 13 nM (with LOQ ≈ 39 nM) and high sensitivity (30.6 ± 2.7 μA cm^−2^ μM^−1^), while reporting minimal interference from other common plant volatiles [22]. Compared to direct oxidation approaches, cascade biocatalysis improves chemical discrimination because the signal is gated by enzyme–substrate compatibility rather than only by overlapping redox potentials. The trade-off is practical: enzyme layers introduce storage constraints, activity drift, and batch-to-batch variability that can dominate performance outside controlled laboratory conditions. For on-farm deployment, stabilization strategies (crosslinking chemistry, protective polymers, lyophilized reagents, replaceable cartridges) become as decisive as the underlying electrochemistry.

Beyond MeSA, electrochemical VOC sensing can also focus on phenolic volatiles that are prominent in plant emissions. Fang and Ramasamy reported a tyrosinase-based electrochemical biosensor for p-ethylphenol using a tyrosinase-modified CNT electrode and amperometric transduction, achieving a sensitive response across a micromolar range (reported linear working range up to ~100 μM in their study) with low detection limits [36]. This phenol-oxidase strategy is analytically appealing because it offers catalytic amplification and can be adapted to multiple phenolic targets. The limitation is again interpretability: p-ethylphenol can originate from diverse biochemical routes and microbial activities. Thus, while the platform is valuable for quantifying phenolic VOC burdens, assigning the signal to a specific crop disease requires either (i) well-justified biomarker panels or (ii) coupling with contextual metadata (cultivar, weather, agronomic operations) and classification models trained on real field datasets.

A complementary direction shifts the emphasis from “one target VOC” to classes of volatiles that collectively encode plant status. Umasankar, Rains, and Ramasamy performed electroanalytical characterization of green leaf volatiles on gold electrodes, cis-3-hexenol, cis-hexenyl acetate, and hexyl acetate using CV, DPV, and amperometric i–t methods, reporting sub-micromolar detection limits (~0.3–0.6 μM by i–t at S/N = 3) and quantifying electro-kinetic parameters under hydrodynamic conditions [37]. Importantly, they framed these measurements in the context of discriminating synthetic mixtures that mimic healthy vs. infected plant volatile profiles. The critical insight here is that many GLVs are not “pathogen products” but host-derived signals tied to membrane lipid oxidation and tissue status. Therefore, GLV electroanalysis is more naturally suited to pattern-based monitoring (early deviation from baseline) than to pathogen identification.

**Figure 6:**
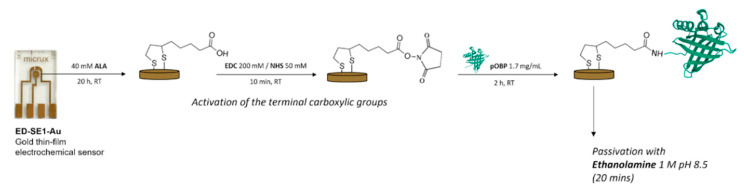
Schematic representation of surface derivatization and functionalization processes [38].

Finally, electrochemical VOC sensing can be implemented with recognition elements borrowed from olfaction, which is conceptually closer to how volatile cues are handled in nature. Calabrese and colleagues [38] constructed a non-Faradaic impedimetric biosensor by covalently immobilizing an odorant-binding protein (pOBP) onto a SAM-functionalized gold electrode and detected VOC binding as changes in interfacial impedance (Figure 6). They demonstrated detection of hexanal, trans-2-hexen-1-ol, and 1-octen-3-ol (notable spoilage- and stress-related volatiles) in the 0.1–10 μM range, including preliminary tests in contaminated air. For crop disease monitoring, this approach is attractive because impedance transduction can operate without requiring the analyte to be electroactive. The main challenge is calibration robustness: impedance is sensitive to humidity, nonspecific adsorption, and electrode-to-electrode variability, so translating “impedance shift” into reliable disease classification demands stringent controls and preferably internal referencing.

Taken together, these cases show that electrochemical VOC sensing for crop disease diagnosis is strongest when positioned as a front-line screening layer rather than a definitive diagnostic. Direct electrooxidation platforms (metal-oxide SPCEs; AuNP–SPCE) can be low-cost and fast, but they face selectivity limits when multiple VOCs share similar electrochemical windows. Enzymatic and protein-based interfaces enhance chemical discrimination (and can enable detection of non-electroactive VOCs), yet they impose stability and manufacturability constraints that are often under-discussed relative to “best-case” LOD values. A pragmatic architecture for early field diagnosis is therefore a tiered workflow: VOC electrochemical sensing flags abnormal plant status and prioritizes sampling locations, while genosensors or immunosensors provide confirmatory pathogen identity when needed. This division of labor aligns the strengths of VOC sensing (speed, noninvasiveness, area coverage) with the strengths of molecular assays (specificity, etiological resolution) and avoids over-claiming disease specificity from single-marker volatiles.

## 3. Critical Analysis of Performance Metrics and Limitations of Current Platforms

While electrochemical biosensing platforms have demonstrated remarkable performance in laboratory settings, their practical application in agricultural settings is limited by several key factors, including matrix interference, stability, cost, and standardization. A critical analysis of the performance metrics of current platforms reveals both their strengths and their inherent limitations.

### 3.1. Performance Metrics: Sensitivity, Specificity, and Speed

The sensitivity of electrochemical biosensors is a key performance metric, as it determines the ability to detect pathogens at early stages of infection, when pathogen concentrations are very low. As revised, Table 2 compares representative electrochemical biosensors in a target-dependent manner, grouping studies by viral, bacterial, fungal/oomycete, and fungal-associated volatile targets. This structure provides a more meaningful comparison of analytical performance within diagnostically related targets. In addition, the time column has been revised to overall assay time, which refers to the full analytical workflow, including sample incubation, amplification or labeling steps where applicable, washing or separation operations, and the final electrochemical readout. This distinction avoids overestimating practical speed when only the instrumental measurement time is reported. Within these representative reports, genosensors and aptasensors generally achieved lower LODs, largely due to nucleic acid amplification, high-affinity target recognition, or signal-amplifying assay architectures. For example, the isothermal genosensor developed by Lau et al. [9] achieved an LOD of 15 copies of *P. syringae* DNA, which is significantly lower than the LOD of conventional PCR (1500 copies). Similarly, the aptasensor developed by Krivitsky et al. [21] achieved an LOD of 100–200 soybean rust spores per cm^2^, which is sufficient to detect early airborne infections.

Specificity is another critical performance metric as it determines the ability of the sensor to distinguish between the target pathogen and other non-target pathogens or interfering substances in the crop matrix. Most electrochemical biosensors exhibit high specificity due to the high affinity of biorecognition elements (antibodies, aptamers, or nucleic acid probes) for their targets. For example, the immunosensor developed by Karimzade et al. [40] exhibited no cross-reactivity with other viral proteins, while the genosensor developed by Lau et al. [9] showed no signal when tested with unrelated pathogens *Botrytis cinerea* and *Fusarium oxysporum* f. sp. *conglutinans*. However, some sensors may exhibit cross-reactivity with closely related pathogen species, particularly when using antibodies that recognize conserved epitopes.

Detection time is a clinically and agronomically relevant metric, but it should be interpreted carefully because published studies do not always define it in the same way. In electrochemical biosensing, the reported value may refer either to the electrochemical signal acquisition step itself or to the total assay time, which can additionally include sample preparation, target capture, incubation, washing, and labeling. This distinction is especially important for immunosensors. Their apparently short detection times arise because antigen–antibody recognition occurs directly at the electrode surface and the electrochemical readout, especially by chronoamperometry or pulse voltammetry, and are usually completed within seconds to minutes. Fast response can be further supported by nanostructured conductive interfaces that accelerate interfacial electron transfer and by enzyme labels that amplify the measurable current. However, the total workflow may still be longer than the readout step alone. For instance, the portable *Xanthomonas oryzae* pv. *oryzae* immunosensor of Razali et al. [41] delivered field results within 200 s on the device, but its laboratory assay format also involved 1 h antigen incubation and 30 min HRP-labeled secondary antibody incubation before measurement. By contrast, label-free immunosensors reduce reagent-handling complexity because they do not require a secondary labeled recognition step, although antigen binding itself may still require tens of minutes; for example, Karimzade et al. [40] optimized the CP-BNYVV immunosensor at an antigen incubation time of about 50 min. In comparison, nucleic acid genosensors often require longer total assay times because amplification or hybridization steps are included, whereas VOC sensors and airborne spore aptasensors can be faster because they rely on direct chemical oxidation or rapid surface binding after collection. The 2 min soybean rust aptasensor of Krivitsky et al. [21] is a representative example of this latter category. Therefore, detection time should be compared across platforms only when the reported value refers to a similar stage of the assay workflow.

### 3.2. Limitations: Matrix Interference, Stability, and Cost

Despite their impressive performance metrics, electrochemical biosensors face several limitations that hinder their widespread adoption in agricultural settings, including matrix interference, limited environmental robustness, cost, and poor reproducibility. Reproducibility is a particularly important but sometimes underemphasized limitation because sensor responses can vary substantially between nominally identical devices due to differences in electrode surface area and morphology, probe immobilization density, nanomaterial distribution, batch-to-batch fabrication, and background fluctuations in complex sample matrices. As noted in the broader electrochemical sensing literature, conventional single-signal electrochemical sensors are especially vulnerable to such variability, which has motivated the development of ratiometric strategies and more standardized fabrication routes to improve measurement consistency. In practical crop-diagnostic settings, this means that an ultralow limit of detection reported for a single optimized device does not necessarily translate into reliable quantitative performance across multiple electrodes, production batches, or field operators. One of the most significant limitations is matrix interference from crop samples, which can include plant sap, soil particulates, phenolics, polysaccharides, pigments (e.g., chlorophyll), and endogenous redox-active metabolites that distort electrode kinetics and/or compromise biorecognition. In practice, this interference manifests as non-specific adsorption (biofouling), altered double-layer capacitance, and redox “background” drift, effects that are often understated when assays are optimized in buffered standards but later transferred to crude plant homogenates. For example, a field-oriented electrochemical immunosensor strip for rice bacterial leaf blight (Xoo) showed that sample preparation strongly influenced matrix interference: compared with scissor-cut leaf preparation, ground leaf extracts produced lower chronoamperometric currents, whereas the less destructive scissor-cut method gave lower blank background and higher recovery. This suggests that gentler handling reduced the release of interfering plant constituents, such as particulates, phenolics, and other fouling components, thereby improving signal-to-noise performance in realistic farm samples [41].

A recurring strategy to reduce matrix effects is front-end target capture/separation, but the tradeoff is added workflow complexity. In a disposable microfluidic electrochemical device for Citrus tristeza virus (CTV), Freitas et al. [17] implemented a magneto-immunoassay where antibody- and HRP-decorated magnetic beads first capture the capsid protein from plant extracts and then deliver the complex into a microfluidic electrochemical cartridge for amperometric readout; the authors report successful discrimination of healthy vs. infected citrus samples with agreement to ELISA. Conceptually, this bead-based “clean-up” is powerful because it physically removes a portion of interfering sap constituents before electroanalysis; however, it also introduces bead handling, wash steps, and enzyme substrate management that can be fragile outside controlled conditions. A similar logic appears in microfluidic immunosensing for *Botrytis cinerea* in fruit tissues, where Fernández-Baldo et al. [25] used micromagnetic beads coupled to SPCEs in a microfluidic format and reported detection in fruit tissues, including asymptomatic fruits, suggesting that structured sample processing can partially offset complex tissue matrices. Yet these microfluidic/MB approaches implicitly shift “matrix robustness” from the electrode interface to the assay protocol, meaning operator variability becomes a dominant error source unless the workflow is tightly packaged and standardized.

Matrix interference is even more acute for nucleic acid targets because plant tissues and crude extracts often contain amplification inhibitors, including polysaccharides, polyphenols, pectin, and other secondary metabolites, which can reduce polymerase efficiency and contribute to false negative results; this problem has been explicitly noted in plant virus diagnostics, especially for inhibitor-rich woody tissues such as grapevine and pome fruit hosts [43]. More recent genosensing work is beginning to acknowledge this explicitly through [9] validation in real total RNA matrices: Lobato et al. [20] developed a label-free impedimetric genosensor for Citrus bark cracking viroid (CBCVd) in hop, highlighting that the study was carried out using real total RNA hop samples and presenting selectivity testing against interferents. This is a meaningful step toward realism: when “interferents” are tested in the same extraction background, the reported selectivity is closer to what growers need. The critical gap, however, is that most papers still provide limited transparency on extraction yield variance, inhibitor carryover, and between-field batch effects, factors that frequently dominate error in early infection stages where nucleic acid levels are lowest.

Stability is another major limitation, as electrochemical biosensors often exhibit reduced performance when exposed to harsh environmental conditions, such as high temperatures, humidity, or UV radiation, which are common in agricultural settings. Stability is not merely a shelf-life question; it is also about how quickly the sensing interface degrades under intermittent wetting/drying cycles, dust exposure, and biofouling from repeated contact with plant fluids. The label-free immunosensor for CP-BNYVV reported retention of ~90% activity after storage at 4 °C for several weeks, which is a meaningful indicator of shelf-life and supports the feasibility of refrigerated storage or transport in insulated containers. However, refrigerated shelf-life alone does not fully establish field robustness, because practical deployment may still involve temperature excursions, humidity/condensation exposure, and performance drift during on-site handling and measurement [39]. In contrast, non-biological recognition layers can ease storage constraints: portable paper-based molecularly imprinted sensors avoid fragile biological receptors and are often easier to store and transport than antibody- or nucleic-acid-based systems [44]. However, better storage stability does not eliminate all practical problems. In molecularly imprinted sensors, the recognition cavities may be partly distorted during handling or mechanical stress, and complex field samples may contain non-target particles or abundant macromolecules that bind non-specifically or compete for the same sites. As a result, a sensor may remain physically stable during storage while still losing selectivity in real plant samples. In other words, “stable” can mean stable to temperature/humidity but not necessarily stable to selectivity erosion in complex matrices.

Cost is also a significant barrier to widespread adoption, particularly for small-scale farmers in developing countries. While electrochemical biosensors are generally cheaper than laboratory-based methods, total cost-of-use is shaped by consumables (electrodes, reagents), sample preparation, training, and failure rates in real matrices. The rice BLB electrochemical strip work illustrates this tension: disposable SPCE-based formats can approach field practicality, but per-test costs may still compete directly with ELISA, depending on local supply chains and whether the biosensor requires enzyme labels and multiple antibodies [41]. On the other end of the spectrum, the TMV paper-based MIP sensor reports an extremely low unit cost (on the order of cents) and minimal reliance on biological reagents, which is attractive for high-throughput screening in low-margin agriculture [44]. However, cost minimization often correlates with reduced analytical metadata (fewer controls, less quantitative calibration infrastructure, weaker traceability to reference methods) unless the platform is paired with rigorous standardization and periodic cross-checking against gold standards (PCR/ELISA). Disposable microfluidic immunoassays for CTV argue for “low-cost diagnosis” and show ELISA agreement, but they still rely on antibodies, magnetic beads, and enzymatic chemistry costs that may not fall proportionally with electrode printing unless mass manufacturing and reagent stabilization (e.g., lyophilization) are solved [17].

## 4. Controversial Perspectives on Field Application of Electrochemical Biosensors

The field application of electrochemical biosensors for crop pathogen detection is a subject of ongoing debate among researchers, with some arguing that these sensors have the potential to revolutionize crop disease management, while others raise concerns about their practical viability in real-world agricultural settings.

### 4.1. Advantages of Field Application

Proponents of electrochemical biosensors argue that their portability, speed, and low cost make them ideal for on-site detection in agricultural settings, enabling rapid disease-management decisions and reducing the spread of pathogens [6]. For example, Razali et al. [41] demonstrated that an electrochemical immunosensor strip for rice bacterial leaf blight could be integrated with an Android-based portable device for on-site testing in hotspot fields, where the readout was displayed and could be stored for remote access; importantly, the platform reportedly detected infection as early as 15 days after transplanting (DAT), before obvious symptoms, which could shift control from reactive spraying to earlier containment actions. Similarly, Krivitsky et al. [21] reported a portable electrochemical strategy aimed at early warning via air surveillance: soybean rust urediniospores were collected at high flow rates and then detected electrochemically using a specific aptamer, framing “field detection” not as leaf testing but as epidemiological monitoring to anticipate outbreaks rather than confirm visible disease.

**Figure 7:**
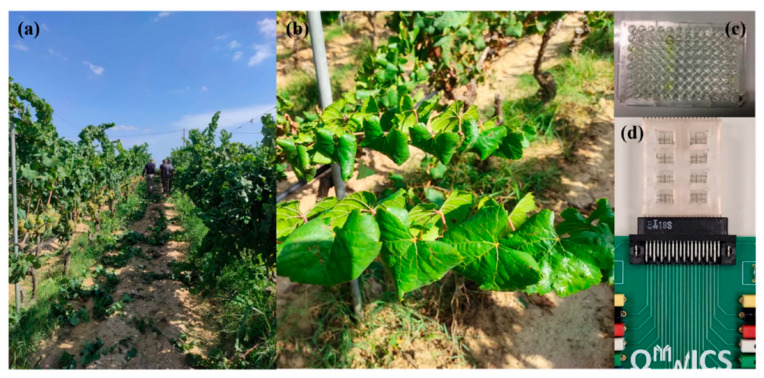
Grapevine leafroll disease and grapevine fanleaf disease diagnosis by ELISA and lab-on-a-chip (LOC) assays to detect Grapevine leafroll-associated virus 3 (GLRaV-3) and Grapevine fanleaf virus (GFLV). (**a**,**b**) Grapevines affected by GLRaV-3; (**c**) ELISA assay with diluted virus sources (1:3, 1:5, 1:10, 1:20, 1:50 and 1:100); (**d**) developed platform for portable, on-field LOC assays, with the sensor inserted in the PCB platform, ready to be connected to the potentiostat. Tests were carried out on different dilutions (1:10, 1:20, 1:50, 1:100 for GLRaV-3 or 1:5, 1:10, 1:20, 1:50, 1:100 for GFLV [47].

Beyond these examples, several recent platforms illustrate how “field applicability” is increasingly engineered as an end-to-end workflow, rather than merely shrinking a benchtop sensor [12]. A notable direction is the integration of mechanically robust sampling interfaces with portable electronics [45]: Ece et al. [46] described an in-field plant pathogen detection concept that uses three-dimensionally printed microneedles for rapid plant sampling coupled to a portable platform, explicitly targeting the bottleneck that many electrochemical assays still rely on laboratory-style extraction steps that are impractical on farms. In parallel, “device portability” is being paired with operator-independent readout and multiplexing for large-scale surveillance. Buja et al. [47] developed a lab-on-a-chip electrochemical impedance platform for grapevine viruses (GLRaV-3 and GFLV) interfaced with portable electronics; they emphasized dose-dependent calibration, multi-channel measurement, and performance benchmarking versus ELISA (Figure 7), positioning the system for field screening programs where throughput and standardized decision thresholds matter as much as analytical sensitivity.

Field use is also motivated by the practical need to detect viruses at very low titers during asymptomatic phases, where centralized testing can be too slow for containment [16]. Rezaei et al. [19] reported ultrasensitive electrochemical immunoassays for tomato brown rugose fruit virus (ToBRFV), including both direct and sandwich formats on a nanoporous gold electrode; the work highlights a common field trade-off: the direct assay prioritizes procedural simplicity (fewer steps, faster execution), whereas the sandwich assay improves sensitivity via enzymatic amplification but increases reagent dependence and workflow complexity—two factors that directly influence whether “portable” assays remain reliable under farm conditions [48]. Complementing antibody-driven methods, imprinting-inspired recognition has been explored for more robust receptor formats [14]: Singh et al. [49] presented a miniaturized electrochemical sensor for bean pod mottle virus using virus-specific nanocavities (molecular imprinting-like cavities within a polymer matrix), aiming to reduce reliance on biological reagents that may be fragile in hot, dusty, or poorly refrigerated environments.

Importantly, in the present review, field electrochemical sensing is not discussed as a virus-only application because fungal disease diagnostics also provide relevant and instructive examples [9,12]. Sambasivam et al. [50] described a rapid electrochemical diagnostic workflow for Botrytis gray mold agents (e.g., B. cinerea and B. fabae) using portable screen-printed electrodes and species-discriminating nucleic acid recognition; this agronomic framing emphasizes fast and quantifiable diagnosis to avoid costly overspraying, thereby linking field deployment not only to early detection but also to input optimization.

Proponents also argue that electrochemical biosensors can be used for continuous or near-continuous monitoring of crop health, enabling earlier detection and reducing the need for frequent manual sampling and laboratory analysis. One operational model is “proxy monitoring” through pathogen- or host-emitted volatiles, where sensors act as an early-warning layer before confirmatory molecular tests [51,52]; for instance, bioelectronic nose concepts have been explored for early detection of *Phytophthora cactorum* in strawberries using disease-associated VOC signatures, illustrating how field monitoring can prioritize sensitivity-to-change and spatiotemporal coverage over single-test definitiveness [42]. Continuous monitoring can, in principle, support more selective interventions and reduce unnecessary fungicide inputs, yet this benefit is contingent on linking sensor outputs to validated decision thresholds and spatial disease models, not merely on the existence of real-time data streams [53,54].

A critical comparison across these field-oriented cases reveals that “portability” alone is an insufficient proxy for real-world utility [55]. The strongest field claims emerge when platforms address three coupled constraints [56]: (i) sampling realism (heterogeneous tissues, low pathogen abundance, inhibitors, dust/moisture contamination), (ii) workflow robustness (few steps, low dependence on cold chain and precise pipetting, stable calibration), and (iii) decision actionability (clear thresholds aligned with agronomic actions such as roguing, quarantine, targeted spraying, or intensified scouting). Airborne surveillance of soybean rust spores is powerful for outbreak prediction, but it can disconnect detection from plant-level severity and may require careful interpretation of “spore counts” versus infection probability under current microclimate and cultivar susceptibility [21]. Virus assays that achieve extremely low limits of detection, such as ToBRFV immunoassays, are analytically impressive, but field reliability can be undermined by batch-to-batch electrode variability, nonspecific adsorption in crude extracts, and the need to maintain enzyme/substrate performance across temperature swings [19,57]. Conversely, reagentless or receptor-stable approaches (e.g., nanocavity-based recognition) may improve ruggedness, but they can introduce a different risk: cavity selectivity may degrade with closely related strains, mixed infections, or matrix fouling, requiring stringent cross-reactivity panels that are often under-reported in proof-of-concept studies [49].

Finally, continuous monitoring narratives should be treated cautiously. Real-time sensing does not automatically translate into reduced chemical use; reductions depend on whether monitoring is integrated into decision-support systems that account for disease spatial patchiness, weather-driven infection windows, and the economics of false alarms [54]. In this sense, the most convincing “field application” biosensing platforms are those that (a) explicitly benchmark against incumbent diagnostics under realistic matrices, (b) report operational constraints (time-to-result, power, consumables, storage), and (c) validate how the output changes a management decision. LOC impedance systems aimed at large-scale grapevine screening are conceptually strong because they connect portability to throughput and operator-independent readout, features that matter for certification programs and regional surveillance, yet they still face deployment barriers such as sample preparation standardization and the logistics of running controlled assays outside lab infrastructure [56]. Overall, the literature supports genuine advantages of field electrochemical biosensing, but the field is moving from “sensor demonstrations” toward deployable diagnostic workflows, where the decisive innovations are often in sampling, QA/QC, and decision linkage rather than in sensitivity metrics alone [30,58,59,60].

### 4.2. Challenges of Field Application

Opponents of electrochemical biosensors raise several concerns about their practical viability in real-world agricultural settings, including their stability, sensitivity to matrix interference, and lack of standardization [6]. For example, some researchers argue that the stability of electrochemical biosensors under field conditions is a major concern, as exposure to high temperatures, humidity, and UV radiation can degrade the biorecognition elements and reduce the sensor’s performance [61]. Additionally, the matrix interference from crop samples can affect the accuracy and reliability of the sensor’s results, leading to false positives or false negatives [62].

**Figure 8:**
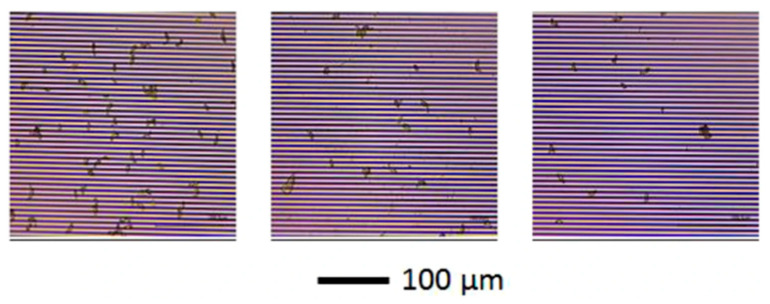
Optical images of ascospores selectively captured by immobilized anti-*S. sclerotiorum* antibodies on SAM-modified IDE surface. Each 3 mm × 3 mm IDE was incubated, using PDMS mask with square wells, with a 50 µL solution containing a suspension of a desired concentration of ascospores in nanopure water [64].

A recurring point in this debate is that many “field” demonstrations still rely on laboratory-like control of reagents, temperature, and sample handling, which can mask stability and interference problems rather than solving them [63]. For instance, an immuno-impedimetric biosensor designed for onsite monitoring of *Sclerotinia sclerotiorum* airborne ascospores (to forecast Sclerotinia stem rot in canola) explicitly frames portability and automation as the key unmet need, yet its analytical correlation is established by incubating interdigitated electrodes with ascospore suspensions in nanopure water under controlled conditions, then linking the impedance shift to captured spore counts (Figure 8) [64]. This is scientifically rigorous for mechanism validation, but it also underscores the practical gap: translating “ascospore-in-water” calibration into a robust field workflow still requires reliable spore trapping, transfer into a measurement-compatible medium, and management of confounders such as dust, pollen, fungal debris, and variable humidity, each of which can alter nonspecific adsorption and interfacial capacitance in non-Faradaic EIS [65]. In other words, the sensor may be portable, while the sampling chain remains the fragile component, an issue that critics often treat as inseparable from “sensor stability” because instability frequently enters through uncontrolled pre-analytics rather than the electrode itself [66].

Matrix effects are equally evident in plant virus sensing, where “simple-to-prepare” extracts can be chemically and rheologically complex (polysaccharides, phenolics, pigments, proteins), and this complexity interacts with both biorecognition and transduction [67]. An impedimetric immunosensor for Plum pox virus (PPV) was reported specifically for detection in plant extracts [68]. Even when such assays succeed analytically, critics note that impedance-based readouts are inherently sensitive to bulk ionic strength, viscosity, and nonspecific surface fouling; thus, small differences in extraction buffer composition, plant cultivar, leaf age, and infection stage can shift the baseline and compress the dynamic range [69]. The practical implication is that a calibration curve built in buffered standards may not be transferable across farms or seasons unless matrix-matched calibration, internal referencing, or standard-addition strategies are built into the protocol, yet these additions raise labor and cost requirements, undermining the simplicity argument often used to justify electrochemical platforms in agriculture [70].

**Figure 9:**
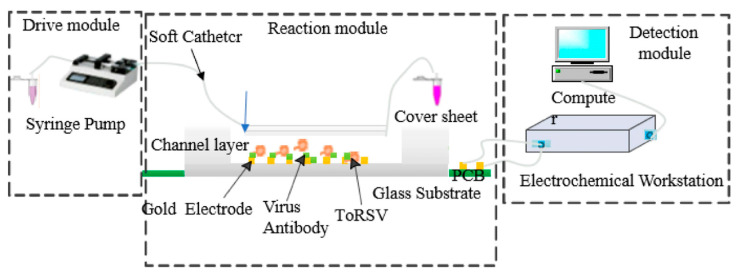
Schematic diagram of the microfluidic impedance detection system [72].

Microfluidic integration is sometimes presented as a countermeasure to matrix interference (by controlling volumes, contact times, and transport), but it can also intensify standardization challenges because performance becomes sensitive to chip-to-chip variation and operating frequency selection [71]. A representative example is a microfluidic impedance sensor for Tomato ringspot virus (ToRSV), where antibodies are immobilized on gold interdigitated array microelectrodes and ToRSV binding is quantified via impedance changes at an “ideal” detection frequency identified experimentally (Figure 9) [72]. While the study reports strong linearity over a defined concentration range, it also illustrates why critics remain cautious: microfluidic architectures add new sources of variance (channel geometry tolerances, bubble formation, surface aging, flow regime) and can make the assay more operator-dependent unless the device is fully standardized and self-calibrating [73]. Moreover, the need to select a specific frequency for optimal discrimination is not merely a methodological detail; it is a reminder that different laboratories may report different “best” conditions, contributing to the broader reproducibility and comparability problem highlighted by opponents [74].

In fungal diagnostics, some electrochemical designs explicitly claim “robust resistibility to complex matrix,” yet this claim itself can be contentious without transparent cross-matrix validation [75]. For example, an ultra-sensitive magnetic-controllable electrochemical biosensor was developed for early diagnosis of rice blast fungus (*Magnaporthe oryzae*) in rice plants, using a fungal chitinase biomarker and a lectin-based recognition strategy with signal amplification [76]. The work is important because it targets pre-symptomatic infection, a central motivation for early warning systems. However, a critical reading reveals that “matrix robustness” can mean different things: resistance to nonspecific adsorption is not the same as resistance to biological confounding (e.g., chitinase-like activity from other fungi, stress-induced plant enzymes, or mixed infections). In real fields, where multiple stresses co-occur, biomarker specificity can drift from the controlled setting, potentially leading to false alarms that erode farmer trust. Therefore, critics argue that the central question is not only whether a biosensor can detect a target at low levels but whether it can maintain decision accuracy when the biological context deviates from the design assumptions.

The stability critique also extends beyond biorecognition lifetimes to include the stability of the measurement baseline under highly variable environmental conditions [77]. Nucleic acid sensors may avoid some cross-reactivity issues seen in immunosensors, but they often reintroduce field fragility via extraction and handling requirements. A graphene-based electrochemical DNA biosensor for rice false smut (*Ustilaginoidea virens*) demonstrates how paper/electrode integration can improve portability and analytical sensitivity for fungal geno-detection [28]. Yet the field bottleneck frequently shifts to nucleic acid preparation (inhibitors co-extracted from plant tissue, variable yield, contamination control), and these steps can dominate the total error budget more than the electrochemical readout itself [78]. From a critical perspective, this creates a paradox: electrochemical detection can be fast and sensitive, but the overall diagnostic system may still behave like a laboratory assay unless upstream sample processing is simplified and standardized [79].

Opponents also argue that the lack of standardization of electrochemical biosensors is a major barrier to their widespread adoption, as different sensors may exhibit varying performance metrics and require different sample preparation methods [80]. For example, the LOD of electrochemical biosensors can vary significantly depending on the type of sensor, the biorecognition element, and the detection method, making it difficult to compare the performance of different sensors. Additionally, the lack of standardized protocols for sample preparation and data analysis can lead to inconsistent results, making it difficult for farmers to interpret the sensor’s data and make informed disease management decisions. Here, the issue is not merely “different LOD numbers,” but the deeper mismatch in what is being quantified and under which measurement conventions: plant virus studies may report mass concentration, particle concentration, or impedance at a chosen frequency; spore monitoring may report spores per mL in a collection medium while agronomic thresholds are defined per cubic meter of air; and fungal biomarkers may be reported as enzyme-linked signals rather than organism load. Without harmonized reporting units, negative-control definitions, matrix-matched validation, and agreed decision thresholds tied to actionable agronomic interventions, high sensitivity alone does not translate into operational utility. Consequently, a persuasive pathway forward is to treat standardization as part of the device design (built-in calibration, reference channels, standardized extraction cartridges, and decision-support outputs), rather than an afterthought left to end users, because in agriculture, usability and trust are often more limiting than analytical capability.

### 4.3. Balancing Advantages and Challenges

The controversy surrounding the field application of electrochemical biosensors highlights the need for further research and development to address the challenges associated with their use in real-world agricultural settings. While proponents argue that these sensors have the potential to revolutionize crop disease management, opponents raise valid concerns about their stability, sensitivity to matrix interference, and lack of standardization. To address these challenges, researchers are developing new materials and fabrication methods to improve the stability and performance of electrochemical biosensors under field conditions, as well as standardized protocols for sample preparation and data analysis [62,81].

A useful way to move beyond this qualitative debate is to interrogate what “field readiness” has actually looked like in concrete plant pathogen case studies and why similar electrochemical principles can produce very different outcomes once crude plant matrices, non-laboratory operators, and variable environments are introduced. For fungal and oomycete-like diseases, several electrochemical nucleic acid platforms demonstrate that high analytical sensitivity is technically achievable, but their workflows quietly reveal where field deployment friction accumulates. For example, a DNA-based electrochemical nanobiosensor targeting *Phytophthora palmivora* (black pod rot in cacao) used sequence-specific probes and electrochemical readout to detect pathogen DNA derived from field isolates, illustrating the core “promise scenario” for plantation surveillance where early diagnosis can plausibly change fungicide timing and pod removal decisions [29]. Yet, the same study logic implicitly depends on reproducible DNA extraction quality and inhibitor tolerance, because cacao pod tissues (polyphenols, polysaccharides) are precisely the kind of matrices that can collapse hybridization efficiency or distort electrochemical backgrounds unless the sample prep step is tightly controlled. In rice blast, an ultra-sensitive magnetic-controllable electrochemical biosensor was reported for *Magnaporthe oryzae* detection directly in rice plant samples, using magnetic manipulation to improve target enrichment and interfacial control, an engineering choice that speaks to a real field constraint: when pathogen biomarkers are dilute and plant extracts are complex, sensor performance becomes dominated by mass-transport, nonspecific adsorption, and the ability to “cleanly” assemble recognition events at the electrode rather than the intrinsic transduction sensitivity [77]. However, magnetic enrichment also introduces operational complexity (magnets, mixing, timing, bead handling) that may be acceptable in a trained extension-lab setting but becomes a reliability liability for decentralized on-farm screening unless the workflow is fully packaged and time-tolerant [82].

A more recent fungal case makes the same tension even clearer. An electrochemical DNA biosensor for *Fusarium oxysporum* f. sp. *cubense* tropical race 4 (banana Fusarium wilt) used dual methylene-blue labeling strategies coupled with structured DNA probes to push sensitivity, reflecting a common trend in electrochemical biosensing: amplification is often achieved through increasingly sophisticated probe architectures rather than through ruggedized sample handling. The critical field question, though, is whether the added molecular complexity buys robustness or merely adds failure modes. Highly engineered oligonucleotide assemblies can outperform simpler probes in buffered conditions, yet they may be more vulnerable to nuclease contamination, temperature excursions, or batch-to-batch immobilization variability, exactly the types of uncontrolled factors that dominate agricultural deployment [83]. In other words, the “advantage” side of the debate (low LOD, fast readout) can be real, but it can also be orthogonal to the bottleneck that determines whether a farmer or field technician gets a correct answer on a humid day from a leaf or stem extract that was not processed perfectly.

Viral diagnostics highlight a different but equally important axis of the controversy: whether electrochemical platforms can match immunoassay convenience without inheriting immunoassay’s vulnerabilities. A disposable microfluidic electrochemical device implementing an ultrasensitive immunoassay for Citrus tristeza virus (CTV) in citrus samples illustrates a pragmatic direction: integrate capture/separation with a confined electrochemical measurement space to reduce operator steps and stabilize assay timing [84]. This “device-level” integration is not just miniaturization; it is an antifouling and standardization strategy, because microfluidic confinement can partially normalize mass transport, reduce exposure to ambient contaminants, and produce more comparable signals between runs. At the same time, immunoassays remain intrinsically exposed to antibody quality, epitope variation, and storage stability; therefore, the strongest argument from skeptics is not that electrochemical readout fails but that biomolecular recognition in agriculture is often the weak link when supply chains, cold-chain realities, and pathogen strain diversity are considered.

The debate becomes even more pointed for emerging and highly regulated crop viruses where false negatives have outsized consequences. A recent electrochemical immunoassay strategy reported attomolar-level detection performance for tomato brown rugose fruit virus (TBRFV) and demonstrated detection in leaf tissue extracts (and even in seed extracts at limited dilution), positioning electrochemical biosensing as a potential biosecurity tool rather than only a farm management accessory. This case is instructive because it implicitly raises the standard for “field suitability”: sensitivity alone is not enough; what matters is validated performance in the real matrices that would be screened (seed lots, leaf sap), with clearly defined dilution limits, controls, and contamination prevention practices. It also underscores why standardization is central to resolving the controversy: without shared benchmarks for matrix panels (healthy leaves across cultivars, mixed infections, pesticide residues, dust), environmental conditions (temperature/humidity), and reporting metrics (decision thresholds, false positive/false negative rates), the literature can look simultaneously “impressive” and “non-translatable.” Supporters and critics can both be right if they are evaluating different endpoints: the supporter emphasizes analytical capability under controlled constraints, whereas the critic evaluates system reliability under heterogeneous field realities [85,86,87].

Taken together, these cases suggest that the most defensible path forward is not simply “better nanomaterials” or “lower LOD” but an explicit shift from sensor-centric optimization to workflow-centric validation. Platforms that embed sample handling (filtration, dilution normalization, inhibitor mitigation), incorporate antifouling surfaces, and enforce procedural timing through device architecture are more likely to survive field conditions than systems that assume laboratory-grade sample quality. Conversely, the field’s skepticism is justified when papers report striking detection limits in spiked buffers but provide limited evidence of robustness across plant species, growth stages, and naturally infected samples. Resolving the controversy therefore requires the community to define what counts as “field performance” and report it consistently; otherwise, electrochemical biosensors will continue to “win” on sensitivity while “losing” on deployability, perpetuating exactly the advantages-versus-challenges stalemate highlighted above.

## 5. Emerging Trends and Future Directions

The future of electrochemical biosensing for crop fungal and viral disease diagnosis will depend less on continuously lowering nominal detection limits and more on solving a small number of practical bottlenecks that determine whether a platform can work reliably under agricultural conditions. Across the literature, the most important of these bottlenecks are matrix tolerance, storage and operational stability, workflow integration, and data interpretation. Recent advances in artificial intelligence (AI), sustainable materials, and connected sensing hardware are valuable not because they are fashionable additions but because they can directly address these specific constraints when implemented in a disciplined and validation-driven manner.

Among these directions, AI-assisted analysis is one of the most promising but also one of the most easily overstated. For electrochemical biosensors, especially impedance-based systems, AI and machine learning can improve signal denoising, artifact suppression, drift correction, and multi-parameter classification from complex readouts [88]. This is particularly relevant for plant diagnostics because crop samples are rarely uniform: cultivar chemistry, growth stage, irrigation history, field microclimate, and tissue heterogeneity can all change the electrochemical background independently of pathogen presence. In such cases, AI can help extract useful patterns from signals that would otherwise appear noisy or inconsistent. However, the critical issue is not whether AI can mathematically fit a dataset but whether the training and validation strategy reflects biological and agronomic reality. If models are trained only on narrowly controlled laboratory datasets, they may perform well in cross-validation while failing under field conditions because of domain shift. This risk is especially acute in plant disease diagnostics, where healthy but stressed plants may generate electrochemical or volatile signatures that resemble infection-related changes. Therefore, AI should be used primarily as a bias-control and decision-support layer rather than as an opaque black-box predictor. The most defensible deployment strategy is to combine electrochemistry-informed feature extraction with lightweight classifiers and explicit uncertainty reporting by, for example, flagging marginal cases for retesting instead of forcing binary outputs [89].

Several examples from the crop pathogen literature illustrate both the potential and the limitations of AI-ready electrochemical platforms. Portable sensing concepts for Tobacco mosaic virus and other viral targets show that miniaturized EIS systems can produce information-rich signals suitable for feature-based classification, but their success depends on selecting frequency regions that are responsive to charge-transfer changes while remaining less sensitive to bulk conductivity fluctuations [90]. In fungal systems, rapid electrochemical sensing of Botrytis spp. and impedance-based monitoring of Phytophthora zoospores suggest that temporal signal evolution, rather than a single endpoint value, may be particularly informative [91]. In principle, this opens an important opportunity for machine learning because time-resolved features may help distinguish true pathogen-related responses from bubbles, particulates, or transient matrix noise. Yet these same systems also raise the bar for dataset curation: labels must be synchronized with orthogonal confirmation methods, and models must be challenged with real irrigation, runoff, sap, or tissue-derived matrices rather than idealized buffers. Thus, future progress in AI-assisted biosensing will depend on stronger validation design, including leave-one-field-out testing, season-to-season transfer evaluation, and negative controls that include abiotic stress conditions as well as non-target pathogens [92].

A second important direction is the use of sustainable or biodegradable sensor materials. This trend is attractive because crop diagnostics is often envisioned as large-scale, disposable, and decentralized; under those conditions, the material footprint of a sensing platform becomes relevant. Paper, cellulose, and other bio-derived supports are therefore increasingly explored as low-cost substrates for electrochemical devices [93]. Their appeal is not limited to environmental considerations. Paper-based platforms can also simplify fluid handling through capillary transport, reduce dependence on pumps and valves, and support low-cost fabrication compatible with field deployment [94]. For instance, paper-based or bio-derived formats have been explored for Xanthomonas-related crop sensing and viral nucleic acid readout, demonstrating that biodegradable supports can contribute simultaneously to portability and affordability [95,96].

However, the current literature also shows that the phrase “biodegradable sensor” must be used carefully. A biodegradable support is not equivalent to a fully biodegradable sensing system. Many electrochemical devices fabricated on paper still rely on carbon inks, metallic nanoparticles, polymeric laminates, adhesives, blocking reagents, or redox-active labels that are not readily degradable, and these additional components often dominate real end-of-life behavior [97]. The same trade-off appears in pathogen-specific examples. Paper electrodes combined with graphene derivatives or other conductive nanomaterials can substantially improve signal quality, as seen in crop-related nucleic acid sensing concepts, but they also increase material complexity and may weaken the sustainability claim if the gain in performance is small relative to the additional material burden [98]. Likewise, pathogen-specific DNA biosensors for diseases such as black pod rot remain attractive candidates for transfer onto cellulose or paper supports, yet real field matrices such as cacao pod extracts introduce particulates, polyphenols, and other inhibitory components that can foul porous materials unless passivation or filtration layers are added, again complicating both degradability and manufacturing simplicity [99,100]. The future direction here is therefore not simply “more paper-based devices” but better whole-system design in which substrate choice, conductive ink composition, reagent storage, barrier layers, and disposal strategy are optimized together.

Reagent logistics form a third critical parameter that links sustainability, portability, and field performance. Several promising electrochemical systems, especially for nucleic acid detection, rely on isothermal amplification or enzyme-assisted signaling. These approaches can reduce dependence on conventional laboratory thermocycling and can dramatically improve sensitivity, but they often introduce new constraints related to enzyme storage, humidity exposure, timed incubation, and cold-chain dependence [101]. This means that genuine field translation requires more than a compact electrode; it requires stable reagent integration within the device architecture. Cartridge-based or paper-assisted storage formats may help, but only if they maintain analytical reliability after realistic handling and environmental cycling. In practical terms, a slightly less sensitive system with superior reagent stability may be more useful in agriculture than a highly sensitive platform that degrades rapidly outside refrigeration [102].

A fourth and equally important direction is the integration of electrochemical biosensors with portable electronics, wireless communication, and IoT-enabled decision workflows. This integration is valuable because field diagnosis is rarely a one-sensor problem. Crop disease management often depends on spatial coverage, repeated measurements, and communication of results to growers or monitoring networks. IoT-compatible electrochemical systems can, in principle, collect data from multiple sensing sites, transmit results to a local gateway or cloud interface, and support timely management recommendations. The rice bacterial leaf blight immunosensor reported by Razali et al. [41] is a useful field-oriented example because it linked portable electrochemical readout with on-site testing in hotspot areas rather than treating portability as a purely instrumental feature. Similarly, disposable microfluidic electrochemical systems developed for *Citrus tristeza* virus emphasize that portability is most meaningful when sample handling, recognition, and readout are integrated into a controlled device workflow rather than distributed across separate manual steps [17]. These examples support a broader point: future crop electrochemical biosensors should be designed as part of a connected diagnostic workflow, not as isolated electrodes.

At the same time, IoT integration should not be confused with genuine deployability. Real-time data transmission does not solve weak matrix robustness, unstable reagents, or poor calibration. Nor does a cloud-connected system automatically produce better agronomic decisions. For connected biosensing to be useful, the measurement itself must already be sufficiently robust, and the output must be linked to actionable thresholds. This is particularly important for disease surveillance, where false positives can trigger unnecessary spraying or quarantine responses, while false negatives can allow outbreaks to spread. Thus, the true value of IoT in this field lies in standardizing data capture, enabling longitudinal monitoring, supporting traceability, and integrating sensor outputs with disease-risk models, rather than merely adding connectivity for its own sake.

Taken together, these developments suggest that the most defensible path forward is a transition from sensor-centric optimization to workflow-centric engineering. Future electrochemical biosensors for crop fungal and viral diseases should be judged less by best-case analytical figures obtained in ideal media and more by whether they can maintain selectivity, stability, and usability across heterogeneous plant matrices and realistic field workflows. In that context, the most critical parameters for future discussion are clear. First, matrix tolerance must be improved through antifouling interfaces, better sample normalization, or controlled pre-processing. Second, reagent and interface stability must be demonstrated under storage and handling conditions relevant to agricultural deployment. Third, system integration should reduce operator dependence by combining sample processing, sensing, and readout within reproducible device architectures. Fourth, AI and digital connectivity should be used to support signal interpretation, quality control, and decision-making, but only under rigorous validation frameworks. If these priorities are addressed together, electrochemical biosensing platforms will be much better positioned to move beyond impressive laboratory demonstrations and toward reliable, scalable, and sustainable crop disease diagnostics.

## 6. Conclusions

In summary, electrochemical biosensing platforms represent a transformative approach for the rapid and early diagnosis of crop fungal and viral diseases, addressing critical limitations of conventional diagnostic methods such as PCR and culturing, which are often time-consuming and laboratory-dependent. The reviewed platforms demonstrate remarkable analytical performance, with immunosensors achieving limits of detection (LODs) as low as 0.3 fg mL^−1^ for Citrus tristeza virus, genosensors reaching detection thresholds down to 15 DNA copies or 0.5 fg µL^−1^ of nucleic acid, and aptasensors capable of identifying 100–200 airborne soybean rust spores cm^−2^ within 2 min. VOC-based electrochemical sensors further enable nanomolar-level detection of pathogen-associated volatiles, such as 35 nmol L^−1^ for p-ethylguaiacol, supporting non-destructive early screening. Despite these impressive metrics, linear ranges spanning several orders of magnitude (10–10^5^ fg mL^−1^), detection times from 2 min to 60 min, and high selectivity against non-target pathogens, field translation remains constrained by matrix interference, reagent stability, and a lack of standardized protocols. Evidence from microfluidic integration, magnetic enrichment, nanoengineered electrodes, and paper-based formats demonstrates that workflow design is as critical as intrinsic sensitivity. Emerging directions, including AI-assisted impedance analysis, biodegradable substrates, nanochannel antifouling architectures, and portable lab-on-chip systems, offer realistic pathways to enhance robustness and scalability. Ultimately, the future of electrochemical crop diagnostics depends not solely on achieving lower LOD values but also on delivering reproducible, cost-effective, and decision-actionable systems capable of operating reliably under heterogeneous agricultural environments.

## Figures and Tables

**Table 1:** Comparison of representative electrochemical biosensing platforms.

Sensor Class	Representative Target	Detection Method	Analyte	LOD	LOQ	Linear Range	Ref.
Immunosensor	Citrus tristeza virus (CTV)	Amperometric sandwich immunoassay in disposable microfluidic device	Capsid protein (CP-CTV)	0.3 fg mL^−1^	NR	1.95–10.0 × 10^3^ fg mL^−1^	[17]
Immunosensor	Fig mosaic virus (FMV)	Label-free DPV immunosensor	Viral nucleocapsid	0.03 nM	NR	0.1 nM–1 μM	[18]
Immunosensor	Tomato brown rugose fruit virus (TBRFV)	Direct and sandwich electrochemical immunoassays on nanoporous gold	Recombinant coat protein	1.14 fg mL^−1^ (direct); 1.06 fg mL^−1^ (sandwich)	NR	10–10^5^ fg mL^−1^	[19]
Genosensor	*Pseudomonas syringae*	RPA-assisted AuNP electrochemical genosensor with DPV readout	Target DNA	15 copies	NR	15–1500 copies	[9]
Genosensor	Citrus Bark Cracking Viroid (CBCVd)	Label-free impedimetric genosensor	Total RNA/target viroid sequence	0.5 fg μL^−1^ (5.5 fmol L^−1^)	NR	NR	[20]
Aptasensor	Soybean rust fungi	Aptamer-based DPV biosensor	Airborne urediniospores	~100–200 spores cm^−2^	NR	NR	[21]
VOC sensor	*Phytophthora cactorum*-associated volatile	CV/DPV/amperometric metal oxide sensor	p-Ethylguaiacol	35–62 nM	NR	NR	[22]
VOC sensor	Stress-/pathogen-associated volatile	Bi-enzyme amperometric sensor	Methyl salicylate	13 nM	39 nM	NR	[23]

**Table 2:** Target-dependent comparison of representative electrochemical biosensors for crop pathogen detection, using overall assay time rather than electrochemical readout time alone.

Target Group	Pathogen/Analyte	Sensor Format	LOD	Linear Range	Overall Assay Time	Ref.
Bacterial	*Pseudomonas syringae* DNA	RPA-assisted electrochemical genosensor	15 copies	15–1500 copies	~60 min	[9]
Fungal	Soybean rust urediniospores	Aptamer-based electrochemical biosensor	~100–200 spores cm^−2^	100–1000 spores cm^−2^	~2 min under optimized collection/detection conditions; not directly comparable to a standardized full sample-to-answer workflow	[21]
Viral	CP-BNYVV	Label-free electrochemical immunosensor	150 fg mL^−1^	0.5–50,000 pg mL^−1^	~50 min incubation + measurement	[39]
Viral	GBNV	GO-based electrochemical immunosensor	5.7 ± 0.7 ng mL^−1^	0.5–150 ng mL^−1^	NR (not explicitly reported as a standardized full workflow time in the primary source)	[40]
Bacterial	*Xanthomonas oryzae* pv. *oryzae* (Xoo)	Electrochemical immunosensor strip	10^2^ CFU mL^−1^	10^2^–10^8^ CFU mL^−1^	~1 h antigen incubation + 30 min secondary antibody incubation + 200 s readout	[41]
Fungal-associated volatile	*Phytophthora cactorum* marker, p-ethylguaiacol	Metal oxide electrochemical VOC sensor	35–62 nM	35–1000 nM	NR (primary source reports electrochemical sensing performance, but not a clearly standardized overall assay time)	[42]

## Data Availability

No new data were created or analyzed in this study.
